# Ivermectin to prevent hospitalizations in patients with COVID-19 (IVERCOR-COVID19) a randomized, double-blind, placebo-controlled trial

**DOI:** 10.1186/s12879-021-06348-5

**Published:** 2021-07-02

**Authors:** Julio Vallejos, Rodrigo Zoni, María Bangher, Silvina Villamandos, Angelina Bobadilla, Fabian Plano, Claudia Campias, Evangelina Chaparro Campias, Maria Fernanda Medina, Fernando Achinelli, Hector Andres Guglielmone, Jorge Ojeda, Diego Farizano Salazar, Gerardo Andino, Pablo Kawerin, Silvana Dellamea, Antonia Cristina Aquino, Victor Flores, Carolina N. Martemucci, Silvina Maria Martinez, Juan Emanuel Segovia, Paola Itati Reynoso, Noelia Carolina Sosa, Mariana Elizabeth Robledo, Joaquina Maria Guarrochena, Maria Mercedes Vernengo, Natalia Ruiz Diaz, Elba Meza, María Gabriela Aguirre

**Affiliations:** 1 Instituto de Cardiología de Corrientes, Bolivar 1334, Zip code, 3400 Corrientes, Argentina; 2 Epidemiology. Ministerio de Salud Pública de la Provincia de Corrientes, Corrientes, Argentina; 3 Hospital de Campaña, Ministerio de Salud Pública de la Provincia de Corrientes, Ministerio de Salud Pública de Corrientes, Corrientes, Argentina; 4 Ministerio de Salud Pública de la Provincia de Corrientes, Corrientes, Argentina

## Abstract

**Background:**

Severe acute respiratory syndrome coronavirus 2 (SARS-CoV2) has changed our lives. The scientific community has been investigating re-purposed treatments to prevent disease progression in coronavirus disease (COVID-19) patients.

**Objective:**

To determine whether ivermectin treatment can prevent hospitalization in individuals with early COVID-19.

Design, setting and participants: A randomized, double-blind, placebo-controlled study was conducted in non-hospitalized individuals with COVID-19 in Corrientes, Argentina. Patients with SARS-CoV-2 positive nasal swabs were contacted within 48 h by telephone to invite them to participate. The trial randomized 501 patients between August 19th 2020 and February 22nd 2021.

**Intervention:**

Patients were randomized to ivermectin (*N* = 250) or placebo (*N* = 251) arms in a staggered dose, according to the patient’s weight, for 2 days.

**Main outcomes and measures:**

The efficacy of ivermectin to prevent hospitalizations was evaluated as primary outcome. We evaluated secondary outcomes in relationship to safety and other efficacy end points.

**Results:**

The mean age was 42 years (SD ± 15.5) and the median time since symptom onset to the inclusion was 4 days [interquartile range 3–6]. The primary outcome of hospitalization was met in 14/250 (5.6%) individuals in ivermectin group and 21/251 (8.4%) in placebo group (odds ratio 0.65; 95% confidence interval, 0.32–1.31; *p* = 0.227). Time to hospitalization was not statistically different between groups. The mean time from study enrollment to invasive mechanical ventilatory support (MVS) was 5.25 days (SD ± 1.71) in ivermectin group and 10 days (SD ± 2) in placebo group, (*p* = 0.019). There were no statistically significant differences in the other secondary outcomes including polymerase chain reaction test negativity and safety outcomes.

**Limitations:**

Low percentage of hospitalization events, dose of ivermectin and not including only high-risk population.

**Conclusion:**

Ivermectin had no significant effect on preventing hospitalization of patients with COVID-19. Patients who received ivermectin required invasive MVS earlier in their treatment. No significant differences were observed in any of the other secondary outcomes.

**Trial registration:**

ClinicalTrials.gov NCT04529525.

**Supplementary Information:**

The online version contains supplementary material available at 10.1186/s12879-021-06348-5.

## Background

Life has changed completely since severe acute respiratory syndrome coronavirus 2 (SARS-CoV-2) was declared a pandemic by the World Health Organization (WHO) on March 11, 2020. Due to coronavirus disease (COVID-19), more than 123,622,286 people have already been infected and more than 2,722,156 have died worldwide [1].

SARS-CoV-2, the COVID-19 etiological agent, is a highly contagious and rapidly spreading virus [2, 3]. The course of the disease can have a varied spectrum of manifestations, from asymptomatic to mild, moderate, or severe pulmonary disease, with multi-organ failure and death of the patient [4].

The scientific community has investigated multiple therapies for the prevention and/or treatment of COVID-19. This spectrum includes immunomodulatory antivirals, therapies with convalescent plasma or hyperimmune equine plasma, anticoagulants, antibiotics, renin angiotensin system inhibitors and glucocorticoids, among others [5–11].

However, none of the aforementioned therapies have had any effect in inhibiting viral replication, nor have they been widely used in non-hospitalized individuals with mild infection. One of the major challenges for the scientific community, is to find an easy-to-administer, low-cost drug with acceptable efficacy to be administered to individuals in home isolation.

Thus, ivermectin has attracted interest since the beginning of the pandemic. It is an antiparasitic drug declared by the WHO as an essential drug for the treatment of certain parasitic infections [12] has also demonstrated antiviral activity against a certain group of viruses [13–15].

In an in vitro study, ivermectin was shown to reduce SARS-CoV-2 virus replication by approximately 5000-fold in the first 48 h [16]. It was from this study, that ivermectin began to be evaluated as a potential treatment and/or preventative for COVID-19, as well as being used off-label in many parts of the world [17–19]. Ivermectin has been shown to be a safe drug for oral administration, even at higher doses than usual and for longer than the standard indications [20].

Current evidence regarding the efficacy of ivermectin for the treatment of COVID-19 patients is still unclear [21, 22], and the WHO recommends its use only for clinical studies [23]. Therefore, the aim of this study is to evaluate the efficacy of ivermectin in preventing hospitalizations in patients with COVID-19 (IVERCORCOVID19) [24].

## Methods

### Trial design and oversight

A randomized, double-blind, placebo-controlled study was conducted in the community between August 19, 2020 and February 22, 2021 in the province of Corrientes, Argentina. This trial was conducted by the Ministry of Public Health of the Province of Corrientes in coordination with the Corrientes Institute of Cardiology “Juana F. Cabral”. It was authorized by the Health Sciences Research Bioethics Committee (HSRBC) of the National University of the Northeast (UNNE) Faculty of Medicine, Argentina (Supplementary Appendix). The study has been supervised by a Steering Committee and a Safety Committee. The trial was performed in accordance with the Declaration of Helsinki and all methods were performed in accordance with the relevant guidelines and regulations. The trial was registered on ClinicalTrials.gov (NCT04529525) on 27/08/2020 and the protocol is available online.

The authors who have designed the protocol and written the manuscript are listed in the Supplementary Appendix. All the authors collected data and vouch for the accuracy and completeness of the data and the adherence of the trial to the protocol, available in the Supplement Appendix. One of the authors (RZ) analyzed the data, and one of the main co-authors (RZ) wrote the first draft of the manuscript. No one other than an author contributed to writing of the manuscript. The study was not sponsored by any industry and none of the authors received any remuneration for conducting this trial. The tablets (ivermectin and placebo) were manufactured at the Corrientes Drug Factory.

### Trial participants

Individuals who met all the following inclusion criteria and none of the exclusion criteria were eligible for inclusion. Individuals were to be over 18 years of age residing in the province of Corrientes at the time of diagnosis with confirmed COVID-19 diagnosis by polymerase chain reaction test (RT-PCR) (CFX96 qPCR, Bio-Rad) for SARS-CoV2 detection in the last 48 h. If they are women of childbearing age, they should be using a contraceptive method of proven efficacy and safety. All individuals were to weigh at the time of inclusion equal to or greater than 48 kg.

Participants were excluded if they were they required current home oxygen use or required hospitalization for COVID-19 at the time of diagnosis or had a history of hospitalization for COVID-19. Other exclusions criteria were pregnant or breastfeeding women, known allergy to ivermectin or the components of ivermectin or placebo tablets, presence of mal-absorptive syndrome, presence of any other concomitant acute infectious disease, known history of severe liver disease, and recent or expected need for dialysis. Concomitant use of hydroxychloroquine or chloroquine or antiviral drugs due to a viral pathology other than COVID-19 at the time of admission was prohibited as was the use of ivermectin up to 7 days before randomization. Individuals with participation in a research study that involved the administration of a drug within the last 30 days were excluded. All participants provided written informed consent. Inclusion criteria are provided in detail in the protocol.

By order of the Ministry of Health of the Province of Corrientes, all patients with COVID-19 were contacted by for tracing purposes. During this telephone contact, eligible individuals were invited to participate in the trial.

Eligible participants were visited at their homes by the researcher to obtain informed consent and undergo randomization. At this visit, trial participants were provided with the treatment kit containing the randomized medication and indicated the corresponding dose and how to take it.

At the day of randomization (day 0), day 3 (± 1 day) and day 12 (± 2 days) an investigator went to the patient’s home where a nasal swab was performed for RT-PCR, vital signs were collected and treatment compliance was assessed. The final visit was considered to be the time at which the patient received the epidemiological discharge of COVID-19 according to the provisions of the Ministry of Health of the Argentine Nation, or the day of death. A follow-up visit was conducted 30 days after the final visit to assess the vital status.

Throughout the study, one of the researchers maintained daily telephone contact with the participants in addition to collecting data related to the patient’s medical history and adverse events.

### Randomization and intervention

Randomization was performed by one of the investigators through the web-based system using randomly permuted blocks in a 1:1 ratio (Supplementary Appendix). The investigator who performed the randomization was not involved in the dispensing of the medication, inclusion, and follow-up of the patients. The rest of the investigators were blinded to the treatment received, as were the patients. Patients were consecutively assigned to the treatment kit in ascending order at inclusion.

Patients who met the inclusion criteria were randomized to ivermectin plus standard of care (SOC) or placebo plus SOC. The SOC was in accordance with the recommendations of the Argentine Ministry of Health. The dose of ivermectin used was the approved dose in Argentina for the treatment of other diseases, such as parasitic diseases, and it was staggered according to weight. Those weighing up to 80 Kg received 2 tablets of 6 mg (mg) each at inclusion and another 2 tablets of 6 mg each 24 h after the first dose (total 24 mg). Those weighing more than 80 kg and up to 110 kg received 3 tablets of 6 mg each at inclusion and another 3 tablets of 6 mg each 24 h after the first dose (total 36 mg). Those weighing more than 110 kg received 4 tablets of 6 mg each at inclusion and another 4 tablets of 6 mg each 24 h after the first dose (total 48 mg). Individuals randomized to placebo received the equivalent number of placebo tablets to the ivermectin weight-based dosage, at baseline and again after 24 h.

### Data collection

The baseline characteristics, concomitant medication and progress of the patients were collected in the clinical history of the patients and then in the database through Google form.

Data related to the patients’ medical history, COVID-19-related symptoms, daily progress and adverse events during their study participation were collected by daily telephone contact. Vital signs, blood samples and nasal swabs were collected by one of the investigators at the patient’s home or at the hospital if the patient was hospitalized at the time of some of the corresponding visits.

In the event that any of the participants required hospitalization, this was performed in a single hospital in the entire province of Corrientes for patients with COVID-19. Hospitalization data were obtained from the digitized clinical history.

### Outcomes

The primary outcome was hospitalization for any reason of patients with COVID-19. This was defined as a stay of at least 24 h in a health institution, in any of its services, at any point from randomization until the end of study visit.

Secondary objectives were time to hospitalization in those who required it, use of invasive mechanical ventilatory support (MVS), time to invasive MVS in those who required it, negative nasal swabs 3 days (± 1) and 12 days (± 2) from study inclusion, dialysis, ivermectin safety (frequency of adverse events), and all-cause mortality. All outcomes were measured from randomization to the EOS visit.

### Statistical analysis

To calculate the sample size, it w as assumed that 10% of the patients required hospitalization, applying a statistical significance level of 0.05 and with a statistical power of 80%. When the trial was designed, there was not enough evidence of the potential benefit of ivermectin in these patients, therefore calculations were based on an estimated odds ratio in the ivermectin arm between 0.3 and 0.5 with the aim of including a total of 500 patients (250 patients in each group).

Continuous variables were expressed as means [± standard deviation (SD)] or medians [with interquartile range (IQR) 25–75] according to their distribution. Categorical variables were expressed as percentages and their 95% confidence interval (CI). Continuous variables were compared in both groups using Student’s *t*-test or the Mann-Whitney test according to their distribution. Categorical variables were compared across groups using the chi-square test.

For analysis of the primary outcome, logistic regression was used to present the odds ratio and the corresponding 95%CI. For secondary outcomes, the logistic regression was used if the secondary outcome was categorical. The Student’s *t*-test or the Mann-Whitney test was used if the variable was continuous and according to its distribution.

In addition, an analysis of hospitalization-free survival was performed using the log-rank test with its corresponding Kaplan-Meier curve and the Cox regression test. The safety point was analyzed using generalized estimating equations, considering that the same patient could have had more than one adverse event.

Subgroup analyses were prespecified according to whether patients were symptomatic or asymptomatic, according to age (< 65 years or ≥ 65 years), and in symptomatic patients according to duration of symptoms prior to inclusion in the study (< 7 days or ≥ 7 days). A prespecified multivariable analysis of the primary outcome was planned (Supplementary Appendix).

Pre-specified interim analyses including 125, 250 and 375 patients were performed to assess the eventual need for early termination of the study according the Haybittle-Peto boundary for which a *p* value < 0.001 was considered significant (Supplementary Appendix). The results of these analyses were reported to the Steering Committee, the Safety Committee and the HSRBC of the UNNE School of Medicine. In all cases, each committee advised continuation of the study as planned.

Patients were analyzed according to the group to which they were assigned during randomization regardless of whether they later received ivermectin or placebo or did not adhere to treatment compliance (intention-to-treat analysis).

Missing data from the primary endpoint were assigned to be considered as hospitalizations, but there were none. A *p*-value < 0.05 was considered significant. Stata software version 16.0 / SE (StataCorp) was used for the analysis.

## Results

**Fig. 1:**
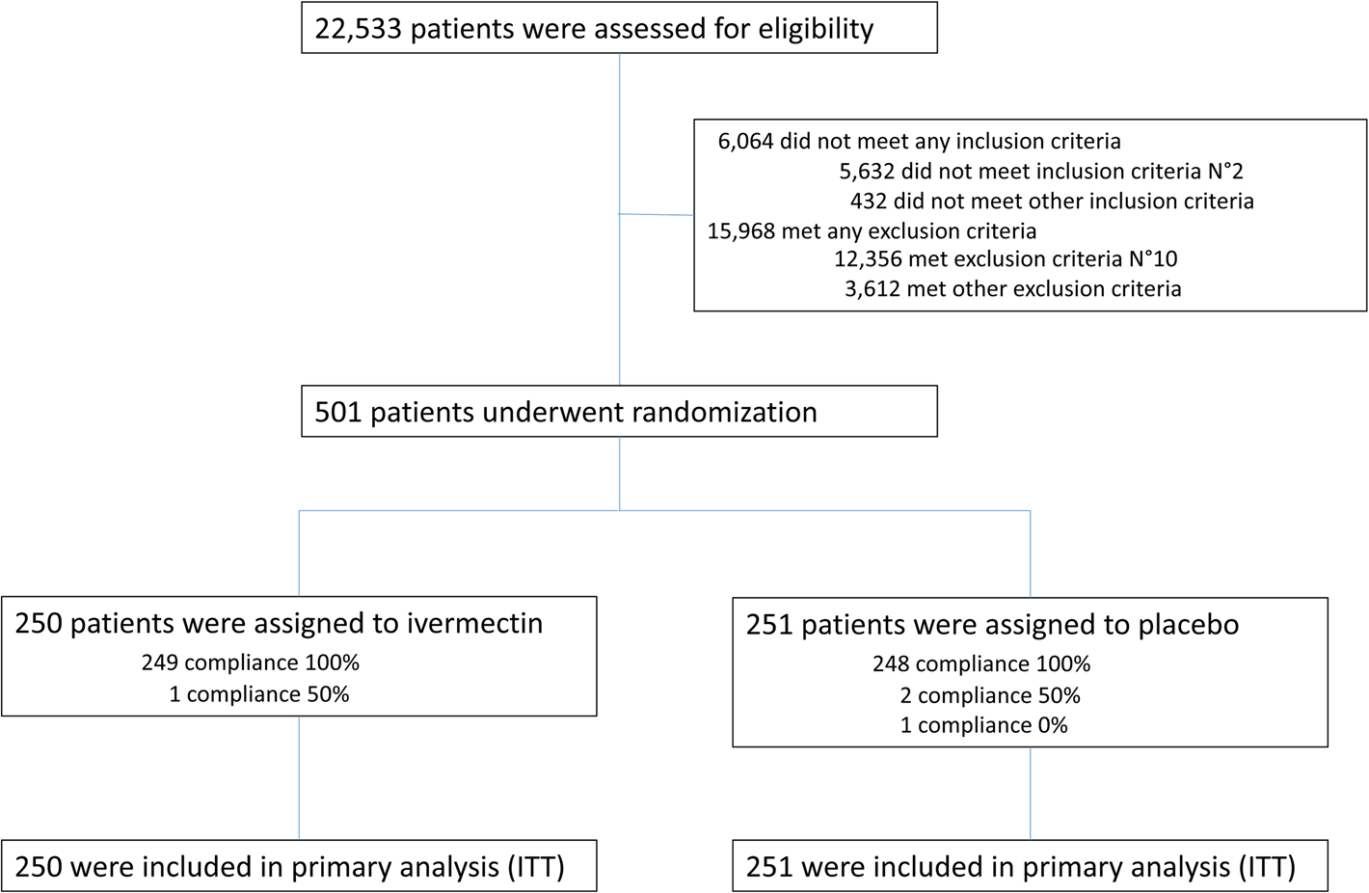
Enrollment and randomization

During the study period, 22,533 SARS-CoV2 positive cases were evaluated for eligibility, of which 501 patients were included (Fig. 1). Two hundred fifty patients were randomized to ivermectin and 251 to placebo. In the ivermectin group, 249 patients had 100% compliance and 1 had 50% compliance. In the placebo group, 248 patients had 100% compliance, 2 patients had 50% compliance and 1 patient had 0% compliance. Considering that an intention-to-treat analysis was performed, all 501 patients were included for the analysis of primary and secondary outcomes. There were no arm crossovers.

Inclusion criteria Number 2: confirmation of COVID-19 diagnosis by polymerase chain reaction test for SARS-CoV2 detection in the last 48 h; exclusion criteria number 10: Use of ivermectin up to 7 days before randomization; ITT: intention-to-treat.

Baseline participants characteristics were similar between the two study groups (Table 1). The mean age was 42.49 years (SD ± 15.51), 237 patients were female (47.31%) and 481 patients were symptomatic (96.01%) for COVID-19. Mean weight was 81.511 kg (SD ± 18.373), 119 patients were hypertensive (23.80%) and 48 (9.62%) were diabetic. The mean number of days from randomization to day 3 (first study swab) was 3.28 (SD ± 0.68); to day 12, (second study swab) was 10.06 (SD ± 1.63) days; and to the end of study visit was 12.43 (SD ± 3.84) days. In the group of symptomatic patients, the median time from symptom onset to inclusion in the study was 4 days (IQR 3–6). The mean ivermectin dose used in the ivermectin group was 192.37 μg/kg/day (SD ± 24.56). All 501 patients completed the 30-day follow-up after the final visit. Baseline characteristics, concomitant treatment prior to study enrollment, laboratory values and physical examination in each group can be seen in Table 1.

**Table 1:** Baseline characteristics

Variables	Ivermectin (*N* = 250)	Placebo (*N* = 251)
Demographic characteristics		
Age years, mean (±SD)	42.58 (15.29)	42.40 (15.75)
< 65 years, N (%)	229 (91.6)	231 (92.03)
Weight Kg, mean (±SD)	81.708 (18.507)	81.313 (18.273)
Dose μg/Kg/day, mean (±SD)	192.37 (24.56)	190.61 (23.93)
Women, N (%)	111 (44.4)	126 (50.2)
Hypertension, N (%)	53 (21.3)	66 (26.3)
Diabetes mellitus, N (%)	21 (8.4)	27 (10.8)
Smoker, N (%)	27 (10.8)	25 (10.0)
Former smoker, N (%)	72 (28.9)	71 (28.3)
Asthma, N (%)	16 (6.4)	20 (8.0)
COPD, N (%)	7 (2.8)	7 (2.8)
Previous myocardial infarction, N (%)	3 (1.2)	6 (2.4)
Previous coronary angioplasty, N (%)	3 (1.2)	1 (0.4)
Previous stroke, N (%)	1 (0.4)	4 (1.6)
Heart failure, N (%)	1 (0.4)	3 (1.2)
Cancer, N (%)	4 (1.6)	2 (0.8)
Previous cancer, N (%)	6 (2.4)	4 (1.6)
Any comorbidity, N (%)	143 (57.66)	149 (59.84)
Symptoms / Swabs		
Symptomatic by COVID-19, N (%)	240 (96.0)	241 (96.0)
Days from symptoms started to inclusion, median (IQR)	4 (3–5)	4 (3–6)
Days from inclusion to swab 1, mean (±SD)	3.26 (0.66)	3.30 (0.71)
Days from inclusion to swab 2, mean (±SD)	9.99 (1.56)	10.13 (1.69)
Previous treatment		
Beta blockers, N (%)	17 (6.8)	21 (8.4)
ACEI, N (%)	13 (5.2)	16 (6.4)
ARB, N (%)	28 (11.2)	36 (14.3)
Aspirin, N (%)	14 (5.6)	19 (7.6)
Statins, N (%)	22 (8.8)	16 (6.4)
Puff inhalation, N (%)	10 (4.0)	11 (4.4)
Corticosteroids, N (%)	12 (4.8)	11 (4.4)
Laboratory values		
Hematocrit %, mean (±SD)	44.63 (5.12)	43.29 (4.90)
Hemoglobin g/dL, mean (±SD)	14.79 (1.79)	14.32 (1.73)
White blood count, mean (±SD)	5931.33 (1952.79)	5550.96 (1778.07)
Platelets /μL, mean (±SD)	232,394.38 (66,306.41)	222,032 (64,476.13)
Creatinine mg/dL, mean (±SD)	0.79 (0.25)	0.81 (0.28)
Urea g/L, median (IQR)	0.30 (0.24–0.37)	0.30 (0.24–0.38)
AST U/L, median (IQR)	27 (21–39)	27 (20–41)
ALT U/L, median (IQR)	31 (18–50)	29 (18–52)
Alkaline phosphatase U/L, median (IQR)	187 (151–234)	187 (153.5–243.5)
Total bilirrubin mg/dL, median (IQR)	0.30 (0.20–0.42)	0.30 (0.20–0.42)
Vital signs		
V#1 Heart rate b/m, mean (±SD)	83.18 (13.64)	82.29 (13.41)
V#1 Oxygen saturation %, mean (±SD)	96.09 (3.16)	96.31 (2.01)
V#1 Axillary temperature °C, mean (±SD)	36.15 (0.84)	36.09 (0.80)
V#2 Heart rate b/m, mean (±SD)	82.05 (12.65)	83.29 (13.35)
V#2 Oxygen saturation %, mean (±SD)	96.13 (2.28)	96.04 (2.42)
V#2 Axillary temperature °C, mean (±SD)	35.99 (0.75)	36.03 (0.77)

*SD* standard deviation; *Kg* kilograms; *μg/Kg/day* micrograms/kilogram/day; COPD: chronic obstructive pulmonary disease; *COVID-19* coronavirus disease 19; *IQR* interquartile range; *ACEI* angiotensin converting enzyme inhibitors; *ARB* angiotensin receptor blockers; *g/dL* grams/deciliter; *μL* microliter; *mg/dL* milligrams/deciliter; *g/L* grams/liter; *U/L* units/liter; *V#* visit number; *b/m* beats/minute; *°C* Celsius degrees; V#1: day 0; V#2: day 3.

### Primary outcome

The trial ended when the last patient who was included achieved the end of study visit.

Of all the individuals who participated in the study, 35 (6.99%) required hospitalization at any point from randomization to their end of study visit. Of these, 14 (5.60%) belonged to the ivermectin group and 21 (8.37%) to the placebo group. There were no statistically significant differences between the two groups [odds ratio 0.65; 95%CI, 0.32–1.31; *p* = 0.227] (Table 2).

**Table 2:** Outcomes from randomization to end of study visit 95% CI: 95% confidence interval; IQR: interquartile range; MVS: mechanical ventilatory support; SD: standard deviation; # total number of events, * All adverse events were non-serious

Outcome	Ivermectin (*N* = 250)	Placebo (*N* = 251)	Odds Ratio (95% CI)	*p* value
Primary				
Hospitalization, N (%)	14 (5.60)	21 (8.37)	0.65 (0.32–1.31)	0.227
Secondary				
Time to hospitalization days (in those who were hospitalized), median (IQR)	3.5 (3–5)	3 (2–5)	–	0.59
Invasive MVS, N (%)	4 (1.60)	3 (1.20)	1.34 (0.30–6.07)	0.70
Time to invasive MVS days (in those who required MVS), mean (±SD)	5.25 (1.71)	10 (2)	–	0.019
Negative nasal swab day 3, N (%)	113 (47.08)	120 (49.79)	0.90 (0.63–1.28)	0.55
Negative nasal swab day 12, N (%)	212 (89.08)	221 (92.47)	0.76 (0.45–1.27)	0.29
Dialysis, N (%)	1 (0.40)	1 (0.40)	1.00 (0.06–16.14)	1
All-cause mortality, N (%)	4 (1.60)	3 (1.20)	1.34 (0.30–6.07)	0.70
Safety (adverse events)#, total (%) *	45 (18.00)	53 (21.11)	–	0.6

### Secondary outcomes

**Fig. 2:**
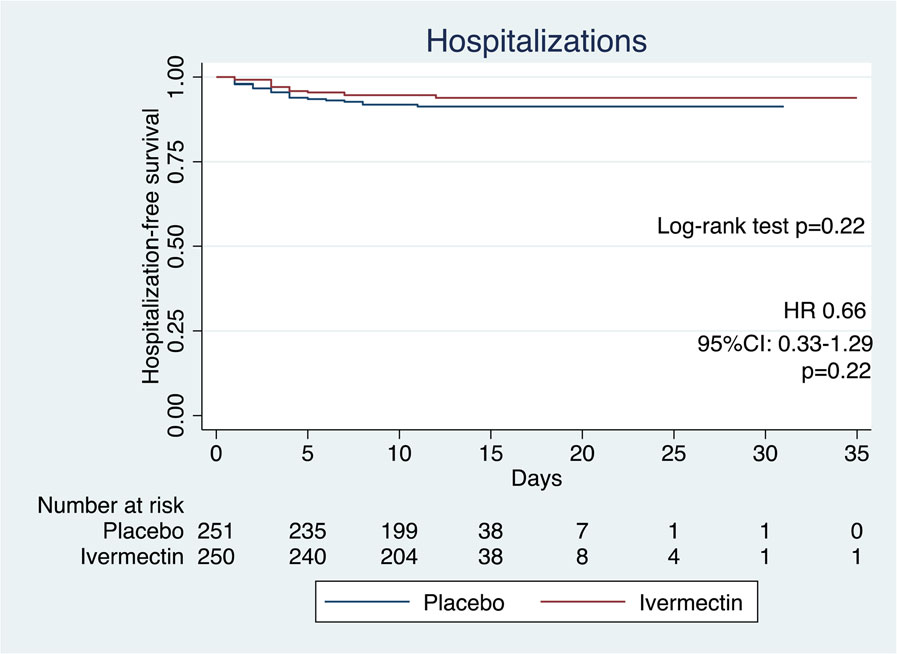
Proportion of hospitalization-free survival time HR: hazard ratio; 95% CI: 95% confidence interval

Secondary outcomes are provided in Table 2. In the group of patients requiring hospitalization, the median time in the ivermectin arm from study enrollment to hospitalization was 3.5 days (IQR 3–5) and in the placebo arm was 3 days (IQR 2–5), with non-statistical difference; *p* = 0.59. (Fig. S2). When analyzing hospitalization-free survival time, there was also no significant difference (hazard ratio 0.66; 95% CI 0.33–1.29; log-rank test *p* = 0.22) (Fig. 2).

Invasive MVS was required in 4 patients (1.6%) in the ivermectin group and in 3 patients (1.2%) in the placebo group with a non-statistical difference (odds ratio 1.34 95% CI 0.30–6.07; *p* = 0.7) (Fig. S3). In this group of patients, the mean time from study enrollment to invasive MVS was 5.25 days (SD ± 1.71) in ivermectin group and 10 days (SD ± 2) in placebo group with statistical difference (*p* = 0.019) (Fig. S4).

When analyzing the RT-PCR results from nasal swabs, the Day 3 (± 1) result was negative for SARS-Cov2 in 113 patients (47.08%) in the ivermectin group and in 120 patients (49.79%) in the placebo group, with a non-statistical difference (odds ratio 0.90; 95% CI 0. 63–1.28; *p* = 0.55) (Fig. S5). RT-PCR at Day 12 (± 2) was negative for SARS-Cov-2 in 212 patients (89.08%) and 221 patients (92.47%) in the ivermectin and placebo groups, respectively (odds ratio 0.76; 95% CI 0.45–1.27; *p* = 0.29) (Fig. S6).

The safety profile, as measured by the need for dialysis, adverse events, and all-cause mortality was similar between groups. Only 2 patients of the 501 study participants required dialysis, 1 in each of the groups (0.4% per group; odds ratio 1.00; 95% CI 0.06–16.14; *p* = 1) (Fig. S7). All-cause mortality was 7 cases (1.40%) in the 501 patients, of which 4 were patients (1.60%) in the ivermectin group and 3 were patients (1.20%) in the placebo group, with a non-statistical difference (odds ratio 1.34 95% CI 0.30–6.07; *p* = 0.72) (Fig. S8). In the follow-up period between the last visit and 30 days thereafter, no new deaths were recorded.

Finally, when analyzing the ivermectin safety point in terms of its adverse events, no per protocol serious adverse events were observed during the study. Non-serious adverse events occurred in 79 patients (15.77%) of the 501 participants, with a total of 98 non-serious adverse events distributed in 45 events (18.00%) in the ivermectin group and 53 events (21.11%) in the placebo group (*p* = 0.6) (Fig. S9 and Table 3). None of the patients discontinued study medication because of adverse events.

**Table 3:** Description of non-serious adverse events

	Ivermectin (*N* = 250)	Placebo (*N* = 251)
Non-serious adverse event, N (%)	45 (18)	53 (21.11)
Perianal abscess, N (%)	0 (0)	1 (0.4)
Anosmia, N (%)	3 (1.2)	1 (0.4)
Asthenia, N (%)	0 (0)	1 (0.4)
Headache, N (%)	1 (0.4)	5 (2)
Diarrhea, N (%)	6 (2.4)	8 (3.2)
Dysgeusia, N (%)	1 (0.4)	0 (0)
Dyspnea, N (%)	1 (0.4)	3 (1.2)
Back pain, N (%)	0 (0)	1 (0.4)
Chest pain, N (%)	1 (0.4)	0 (0)
Worsening muscle pain, N (%)	1 (0.4)	0 (0)
Epistaxis, N (%)	0 (0)	1 (0.4)
Fatigue, N (%)	0 (0)	1 (0.4)
Fever, N (%)	4 (1.6)	4 (1.6)
Stomach flu, N (%)	0 (0)	1 (0.4)
Dizziness, N (%)	1 (0.4)	0 (0)
Myalgia, N (%)	3 (1.2)	0 (0)
Nauseas, N (%)	1 (0.4)	4 (1.6)
Pneumonia, N (%)	16 (6.4)	8 (3.2)
Odynophagia, N (%)	0 (0)	1 (0.4)
Palpitations, N (%)	1 (0.4)	0 (0)
Rash, N (%)	0 (0)	2 (0.8)
Dry cough, N (%)	0 (0)	7 (2.8)
Urticaria, N (%)	1 (0.4)	2 (0.8)
Vomiting, N (%)	0 (0)	1 (0.4)
Arthralgia, N (%)	1 (0.4)	0 (0)
Sciatica, N (%)	0 (0)	1 (0.4)
Progression of dyspnea, N (%)	1 (0.4)	0 (0)
Abdominal pain, N (%)	1 (0.4)	0 (0)
Foot trauma, N (%)	1 (0.4)	0 (0)

### Subgroup analysis

When analyzing the prespecified subgroups, no significant differences were found in hospitalizations (Fig. S10-S15 and Table S1-S6).

## Discussion

In this trial, ivermectin treatment in patients with mild or moderate COVID-19 had no significant effect on preventing hospitalization of patients with COVID-19. In the secondary end points, no significant differences were observed except for the time elapsed from hospitalization to invasive MVS, where patients who received ivermectin required it significantly earlier.

To our knowledge, this is the first randomized, double-blind, placebo-controlled, peer-reviewed study for publication in which the efficacy of ivermectin in preventing hospitalizations was evaluated [25–27]. In a meta-analysis that included 629 patients from 4 studies comparing ivermectin with placebo, it was observed as a secondary outcome that ivermectin was associated with clinical improvement in patients. From this meta-analysis it is noted that the evidence quality of the included studies is debatable [28]. A report from the Pan American Health Organization (PAHO) was recently published on the different therapies for the treatment of COVID where in a meta-analysis that included 4837 patients there was no significant reduction in hospitalizations of patients with mild to moderate COVID-19 and who have been treated with ivermectin [29]. These results are in agreement with those found in IVERCORCOVID19 but it is noteworthy that the studies included in these meta-analysis have methodological limitations that make their quality also debatable.

In line with the results of our trial, a randomized, double-blind study has recently been published. This study analyzed 398 patients according to whether they had received ivermectin or placebo. No significant differences were observed in the primary outcome of symptomatic improvement [22].

The fact that no significant differences were found in the primary end point of hospitalizations in this study may be due to different factors. The first is that ivermectin is not effective in this group of patients to prevent hospitalizations. The second is that the IVERCORCOVID19 trial is underpowered because the hospitalization rate was lower than expected when performed in the sample size calculation, as well as the fact that an ambitious reduction of 50–70% was estimated of primary end point. Thirdly, the dose of ivermectin adjusted to the weight of the patients was low, which on the one hand could corroborate that these doses are not effective, but alternatively could provide the opportunity to study the efficacy of higher doses of ivermectin.

There were no significant differences regarding negative swabs at 3 ± 1 days and 12 ± 2 days with the use of ivermectin. These results differ from those found in a retrospective study in which the median SARS-Cov-2 viral clearance was 4 days in the ivermectin group and 15 days in the placebo group [30]. Likewise, the results also differ from those found in a prospective study of 72 patients in which the use of ivermectin (12 mg once daily for 5 days) was associated with a significant reduction in viral clearance of 3 days compared to placebo (9.7 vs. 12.7 days) [31]. However, they agree with a randomized, double-blind pilot study in which ivermectin administration was not associated with an increase in viral clearance at 7 days [32]. The non-significant trend in reduction in hospitalizations of patients in the ivermectin group is not shown in the secondary end point of negative nasal swabs. This could be in accordance with the hypothesis that ivermectin protects the host cell without directly attacking SARS-CoV-2 which, fully or partially, could be maintained in the host’s body, but with less virulence [16]. This hypothesis should be confirmed with future research studies.

Although no significant differences were observed in the use of invasive mechanical ventilatory support, patients in the ivermectin group required it 4.75 days earlier than those receiving placebo. Although only 7 patients out of the total number of study participants required invasive mechanical ventilatory support, this difference in the ivermectin group in terms of its earliness was statistically significant. This could raise different and disparate hypotheses, such as that patients who received ivermectin required more of this support due to the drug, that these patients who received ivermectin and required the support had more severe conditions, or due to chance because of the small number of patients who presented this event.

As for mortality, there were no significant differences between the two groups. The study was not designed to evaluate this point primarily, and total mortality in the trial was 1.40% (*N* = 7). The aforementioned meta-analysis that included 629 patients had the primary objective of evaluating mortality, which was significantly reduced by 47% [28]. In the PAHO meta-analysis of patients receiving ivermectin, a statistically significant reduction in mortality was not observed with the limitations of the included studies [29]. Due to the low number of mortality events reported in IVERCORCOVID19 and that the study was not designed to primarily evaluate this end point, it cannot be ruled out that the non-significant difference found is due to chance.

When assessing ivermectin safety, no serious adverse events were observed and there were no statistically significant differences in non-serious adverse events compared to placebo. Many observed events could be related to the course of the disease without being able to differentiate whether they were attributed to the study drug. Per protocol, once patients were included in IVERCORCOVID19, the appearance of any new symptom or the worsening of an existing one were considered as adverse events, therefore many of these events could be related to COVID-19 disease. Approximately one non-serious adverse event was reported for every 5 patients.

The initially planned multivariate analysis was not performed since no significant differences were found in the univariate analysis of the primary outcome.

This study has several limitations. Firstly, the percentage of events in relation to the primary outcome was below the estimate, so this trial was under powered.

Secondly, the mean dose of ivermectin was 192.37 μg/kg/day (SD ± 24.56), which is below the doses proposed as probably effective [20, 33]. Thirdly, a middle-aged population was included which, in accordance with the first point raised in this section, had hospitalization events below the 10% set at the time of calculating the sample size. On the other hand, including a population with these characteristics increases the external validity of the study. Consideration could be given to analyzing the efficacy of ivermectin in a population at high risk of hospitalization in future trials. Fourth, blood ivermectin levels were not measured, so we cannot know the bioavailability of the drug in these patients or the blood ivermectin levels that were reached. Lastly, we did not include any scale to determine the severity of the patients who were enrolled. At the time of inclusion in IVERCORCOVID19, the patients did not have hospitalization criteria, therefore, we cannot determine if the population included was mostly with a mild or moderate condition or if there was a similar distribution between both groups.

In the IVERCORCOVID19 trial, in patients with a positive COVID-19 nasal swab by RT-PCR technique in the last 48 h, ivermectin in a staggered dose according to the patient’s weight for 2 days had no significant effect on preventing hospitalization of patients with COVID-19. No significant differences were observed in secondary outcomes such as the time elapsed from study enrollment to hospitalization in those who required it. Additionally, no significant differences were observed in the use of invasive mechanical ventilatory support, the requirement for dialysis, negative nasal swabs at 3 and 12 days after study enrollment, or in all-cause mortality. Patients who received ivermectin required invasive mechanical ventilatory support earlier. The use of ivermectin was not associated with increased adverse events.

## Supplementary Information

**Additional file 1.**

## Data Availability

The datasets used and/or analysed during the current study available from the corresponding author on reasonable request. Steering Committee: Eduardo Farias MD, Eduardo Perna MD and Dr. Jorge Parras MD. Security Committee: Maximiliano Rinas Casullo MD, Luciana Borgo and Margarita Santoro.
