# Supplementary material for: Ivermectin to prevent hospitalizations in patients with COVID-19 (IVERCOR-COVID19) a randomized, double-blind, placebo-controlled trial
Source: BMC Infect Dis. 2021 Jul 2;21:635. doi: 10.1186/s12879-021-06348-5 (PMC8250562; doi:10.1186/s12879-021-06348-5)
Supplement: Supplementary file 1 — Additional file 1. [file 12879_2021_6348_MOESM1_ESM.docx]

Supplementary material

Vallejos J, Zoni R, Bangher M, et al. **Ivermectin to Prevent Hospitalizations in patients with COVID-19 (IVERCOR-COVID19). A randomized, double-blind, placebo-controlled trial.**

**Supplementary Materials Index**

**List of authors** (Page 3)

**Authors’ contributions** (Page 4)

**Figure S1.** Distribution of patients according to randomization (Page 5)

**Figure S2.** Time from inclusion to hospitalization (Page 5)

**Figure S3.** Distribution of patients who required invasive mechanical ventilatory support (Page 6)

**Figure S4.** Time in days from inclusion to invasive MVS (Page 6)

**Figure S5.** Patients with negative nasal swab at 3 days (Page 7)

**Figure S6.** Patients with negative nasal swab at 12 days (Page 7)

**Figure S7.** Distribution of patients who required dialysis (Page 8)

**Figure S8.** All-cause mortality (Page 8)

**Figure S9.** Number of patients with non-serious adverse events (Page 9)

**Pre-specified sub analysis** (Page 9)

**Figure S10.** Distribution of patients according to age (Page 9)

**Table S1.** Baseline characteristics in patients younger than 65 years old (Page 10)

**Table S2.** Baseline characteristics in patients of 65 years old or older (Page 11)

**Figure S11.** Hospitalization according to age (Page 12)

**Figure S12.** Distribution of patients according to symptoms (Page 12)

**Table S3.** Baseline characteristics in asymptomatic patients (Page 12)

**Table S4.** Baseline characteristics in symptomatic patients (Page 13)

**Figure S13.** Hospitalization in patients symptomatic and asymptomatic (Page 15)

**Figure S14.** Distribution in patients symptomatic according to length of symptoms (Page 15)

**Table S5.** Baseline characteristics in patients with less than 7 days symptoms (Page 16)

**Table S6.** Baseline characteristics in patients with equal to or greater than 7 days symptoms (Page 16)

**Figure S14.** Hospitalization in patients symptomatic according to length of symptoms (Page 18)

**List of authors**

Julio Vallejos, Medical Doctor. Instituto de Cardiología de Corrientes. Argentina.

Rodrigo Zoni, Medical Doctor. Instituto de Cardiología de Corrientes. Argentina.

María Bangher, Medical Doctor. Instituto de Cardiología de Corrientes. Argentina.

Silvina Villamandos, Medical Doctor. Instituto de Cardiología de Corrientes. Argentina.

Angelina Bobadilla, Medical Doctor. Epidemiology. Ministerio de Salud Pública de la Provincia de Corrientes. Argentina.

Fabian Plano, Medical Doctor. Hospital de Campaña. Ministerio de Salud Pública de la Provincia de Corrientes. Argentina.

Claudia Campias, Medical Doctor. Epidemiology. Ministerio de Salud Pública de la Provincia de Corrientes. Argentina.

Evangelina Chaparro Campias, Medical Doctor. Ministerio de Salud Pública de la Provincia de Corrientes. Argentina.

Maria Fernanda Medina, Biochemistry. Instituto de Cardiología de Corrientes. Argentina.

Fernando Achinelli, Medical Doctor. Hospital de Campaña. Ministerio de Salud Pública de la Provincia de Corrientes. Argentina.

Hector Andres Guglielmone, Pharmacist. Ministerio de Salud Pública de la Provincia de Corrientes. Argentina.

Jorge Ojeda, Medical Doctor. Hospital de Campaña. Ministerio de Salud Pública de la Provincia de Corrientes. Argentina.

Diego Farizano Salazar, Biochemistry. Hospital de Campaña. Ministerio de Salud Pública de la Provincia de Corrientes. Argentina.

Gerardo Andino, Biochemistry. Central Laboratory. Ministerio de Salud Pública de la Provincia de Corrientes. Argentina.

Pablo Kawerin, Biochemistry. Central Laboratory. Ministerio de Salud Pública de la Provincia de Corrientes. Argentina.

Silvana Dellamea, laboratory assistant. Hospital de Campaña. Ministerio de Salud Pública de la Provincia de Corrientes. Argentina.

Antonia Cristina Aquino, laboratory assistant. Hospital de Campaña. Ministerio de Salud Pública de la Provincia de Corrientes. Argentina.

Victor Flores, laboratory assistant. Hospital de Campaña. Ministerio de Salud Pública de la Provincia de Corrientes. Argentina.

Carolina N Martemucci. Biochemistry. Ministerio de Salud Pública de la Provincia de Corrientes. Argentina.

Silvina Maria Martinez. Biochemistry. Ministerio de Salud Pública de la Provincia de Corrientes. Argentina.

Juan Emanuel Segovia, Biochemistry. Epidemiology. Ministerio de Salud Pública de la Provincia de Corrientes. Argentina.

Paola Itati Reynoso, laboratory assistant. Instituto de Cardiología de Corrientes. Argentina.

Noelia Carolina Sosa, laboratory assistant. Instituto de Cardiología de Corrientes. Argentina.

Mariana Elizabeth Robledo, laboratory assistant. Instituto de Cardiología de Corrientes. Argentina.

Joaquina Maria Guarrochena, Student of Medicine. Corrientes, Argentina

Maria Mercedes Vernengo, Bachelor's Degree of Nutrition. Epidemiology. Ministerio de Salud Pública de la Provincia de Corrientes. Argentina.

Natalia Ruiz Diaz, Biochemistry. Central Laboratory. Ministerio de Salud Pública de la Provincia de Corrientes. Argentina.

Elba Meza, Medical Doctor. Epidemiology. Ministerio de Salud Pública de la Provincia de Corrientes. Argentina.

María Gabriela Aguirre, Study Coordinator. Instituto de Cardiología de Corrientes. Argentina.

JV and RZ are the principal investigators.

**Authors’ contributions:**

Study design: RZ, JV

Writing first draft: RZ

Literature search: RZ, JV, MB, SV

Randomization: JV

Data Collection: SV, MB, ECC, RZ, SD, AA, VF, CNM, SMM, JES, AB, CC, FP, JO, FA, PIR, NCS, MER

Laboratory analysis: FM, DFS, GA, PK, NRD

Drug manufacturing: HAG

Data analysis: RZ

Data uploaded: JMG

Study coordination: RZ, JV, MAG

Supervision of the project: JV, RZ

All the authors read and approved the final manuscript.

**Figure S1.** Distribution of patients according to randomization

**Figure S2.** Time from inclusion to hospitalization in patients who required it. IQR: interquartile range

**Figure S3.** Distribution of patients who required invasive mechanical ventilatory support. OR: odds ratio; CI: confidence interval

**Figure S4**. Time in days from inclusion to invasive MVS. MVS: mechanical ventilatory support; SD: standard deviation

**Figure S5.** Patients with negative nasal swab at 3 days (±1 day) from inclusion. OR: odds ratio; CI: confidence interval

**Figure S6.** Patients with negative nasal swab at 12 days (±2 day) from inclusion. OR: odds ratio; CI: confidence interval

**Figure S7.** Distribution of patients who required dialysis. OR: odds ratio; CI: confidence interval

**Figure S8.** All-cause mortality. OR: odds ratio; CI: confidence interval

**Figure S9.** Number of patients with non-serious adverse events. OR: odds ratio; CI: confidence interval


**Pre-specified sub analysis**

**Sub analysis by age**

**Figure S10.** Distribution of patients according to age

| Variables | Ivermectin (N=229) | Placebo (N=231) |
| --- | --- | --- |
| Demographic characteristics |  | |
| Age years, mean (±SD) | 39.81 (12.73) | 39.64 (13.06) |
| Weight Kg, mean (±SD) | 81.717 (18.827) | 81.485 (18.501) |
| Dose μg/Kg/day, mean (±SD) | 193.10 (24.90) | 191.16 (24.38) |
| Women % | 42.8 (98) | 48.5 (112) |
| Hypertension % | 16.6 (38) | 22.1 (51) |
| Diabetes mellitus % | 5.2 (12) | 8.7 (20) |
| Smoker % | 11.8 (27) | 10.8 (25) |
| Former smoker % | 27.6 (63) | 27.7 (64) |
| Asthma % | 6.1 (14) | 6.9 (16) |
| COPD % | 1.7 (4) | 1.7 (4) |
| Previous myocardial infarction % | 1.3 (3) | 1.7 (4) |
| Previous coronary angioplasty % | 0.9 (2) | 0.4 (1) |
| Previous stroke % | 0.0 (0) | 0.9 (2) |
| Heart failure % | 0.0 (0) | 0.9 (2) |
| Cancer % | 1.3 (3) | 0.4 (1) |
| Previous cancer % | 1.7 (4) | 1.7 (4) |
| Symptoms / Swabs |  | |
| Symptomatic by COVID-19 % | 97.4 (223) | 96.5 (223) |
| Days from symptoms started to inclusion, median (IQR) | 4 (3-5) | 4 (3-6) |
| Days from inclusion to swab 1, mean (±SD) | 3.25 (0.65) | 3.27 (0.69) |
| Days from inclusion to swab 2, mean (±SD) | 9.93 (1.54) | 10.12 (1.69) |
| Previous treatment |  | |
| Beta blockers % | 4.8 (11) | 6.5 (15) |
| ACEI % | 3.9 (9) | 6.5 (15) |
| ARB % | 7.9 (18) | 10.8 (25) |
| Aspirin % | 3.9 (9) | 5.2 (12) |
| Statins % | 6.6 (15) | 4.3 (10) |
| Puff inhalation % | 3.5 (8) | 2.2 (5) |
| Corticosteroids % | 4.4 (10) | 3.9 (9) |
| Laboratory values |  | |
| Hematocrit %, mean (±SD) | 44.84 (5.16) | 43.48 (4.61) |
| Hemoglobin g/dL, mean (±SD) | 14.88 (1.80) | 14.39 (1.65) |
| White blood count, mean (±SD) | 5947.24 (1954.61) | 5629.56 (1790.01) |
| Platelets /μL, mean (±SD) | 234873.36 (67554.50) | 224260 (62085.11) |
| Creatinine mg/dL, mean (±SD) | 0.78 (0.25) | 0.79 (0.22) |
| Urea g/L, median (IQR) | 0.29 (0.24-0.36) | 0.3 (0.23-0.365) |
| AST U/L, median (IQR) | 27 (21-39) | 27 (20-41) |
| ALT U/L, median (IQR) | 32 (18-51) | 29 (18-51) |
| Alkaline phosphatase U/L, median (IQR) | 187.5 (152-233.5) | 186.5 (154-238) |
| Total bilirrubin mg/dL, median (IQR) | 0.30 (0.20-0.40) | 0.30 (0.20-0.42) |
| Vital signs |  | |
| V#1 Heart rate b/m, mean (±SD) | 83.64 (13.61) | 82.32 (13.43) |
| V#1 Oxygen saturation %, mean (±SD) | 96.35 (1.86) | 96.47 (1.76) |
| V#1 Axillary temperature °C, mean (±SD) | 36.16 (0.84) | 36.11 (0.81) |
| V#2 Heart rate b/m, mean (±SD) | 82.40 (12.87) | 83.23 (13.44) |
| V#2 Oxygen saturation %, mean (±SD) | 96.23 (2.31) | 96.20 (2.29) |
| V#2 Axillary temperature °C, mean (±SD) | 36.01 (0.73) | 36.04 (0.75) |

**Table S1.** Baseline characteristics in patients younger than 65 years old.

SD: standard deviation; Kg: kilograms; μg/Kg/day: micrograms/kilogram/day; COPD: chronic obstructive pulmonary disease; COVID-19: coronavirus disease 19; IQR: interquartile range; ACEI: angiotensin converting enzyme inhibitors; ARB: angiotensin receptor blockers; g/dL: grams/deciliter; μL: microliter; mg/dL: milligrams/deciliter; g/L: grams/liter; U/L: units/liter; V#: visit number; b/m: beats/minute; °C: Celsius degrees

| Variables | Ivermectin (N=21) | Placebo (N=20) |
| --- | --- | --- |
| Demographic characteristics |  | |
| Age years, mean (±SD) | 72.70 (4.88) | 74.22 (6.58) |
| Weight Kg, mean (±SD) | 81.619 (14.955) | 79.335 (15.672) |
| Dose μg/Kg/day, mean (±SD) | 184.46 (19.22) | 184.30 (17.26) |
| Women % | 62.0 (13) | 70.0 (14) |
| Hypertension % | 75.0 (15) | 75.0 (15) |
| Diabetes mellitus % | 43.0 (9) | 35.0 (7) |
| Smoker % | 0.0 (0) | 0.0 (0) |
| Former smoker % | 43.0 (9) | 35.0 (7) |
| Asthma % | 10.0 (2) | 20.0 (4) |
| COPD % | 14.0 (3) | 15.0 (3) |
| Previous myocardial infarction % | 0.0 (0) | 10.0 (2) |
| Previous coronary angioplasty % | 5.0 (1) | 0.0 (0) |
| Previous stroke % | 5.0 (1) | 10.0 (2) |
| Heart failure % | 5.0 (1) | 5.0 (1) |
| Cancer % | 5.0 (1) | 5.0 (1) |
| Previous cancer % | 10.0 (2) | 0.0 (0) |
| Symptoms / Swabs |  | |
| Symptomatic by COVID-19 % | 81.0 (17) | 90.0 (18) |
| Days from symptoms started to inclusion, median (IQR) | 4 (2-6) | 4 (3-5) |
| Days from inclusion to swab 1, mean (±SD) | 3.29 (0.72) | 3.58 (0.90) |
| Days from inclusion to swab 2, mean (±SD) | 10.62 (1.75) | 10.30 (1.81) |
| Previous treatment |  | |
| Beta blockers % | 29.0 (6) | 30.0 (6) |
| ACEI % | 19.0 (4) | 5.0 (1) |
| ARB % | 48.0 (10) | 55.0 (11) |
| Aspirin % | 24.0 (5) | 35.0 (7) |
| Statins % | 33.0 (7) | 30.0 (6) |
| Puff inhalation % | 10.0 (2) | 30.0 (6) |
| Corticosteroids % | 10.0 (2) | 10.0 (2) |
| Laboratory values |  | |
| Hematocrit %, mean (±SD) | 42.25 (3.98) | 41.15 (7.36) |
| Hemoglobin g/dL, mean (±SD) | 13.81 (1.43) | 13.47 (2.35) |
| White blood count, mean (±SD) | 5750.00 (1972.72) | 4647.00 (1370.84) |
| Platelets /μL, mean (±SD) | 206500.00 (42938.75) | 196400.00 (85273.18) |
| Creatinine mg/dL, mean (±SD) | 0.88 (0.15) | 1.02 (0.62) |
| Urea g/L, median (IQR) | 0.385 (0.33-0.525) | 0.41 (0.25-0.56) |
| AST U/L, median (IQR) | 28 (22-34) | 35.5 (25.5-47.5) |
| ALT U/L, median (IQR) | 21 (17.5-38.5) | 27.5 (17-56.5) |
| Alkaline phosphatase U/L, median (IQR) | 185 (128-253) | 199.5 (151-289) |
| Total bilirrubin mg/dL, median (IQR) | 0.40 (0.25-0.50) | 0.30 (0.275-0.435) |
| Vital signs |  | |
| V#1 Heart rate b/m, mean (±SD) | 78.38 (13.43) | 82.00 (13.57) |
| V#1 Oxygen saturation %, mean (±SD) | 93.38 (8.62) | 94.42 (3.35) |
| V#1 Axillary temperature °C, mean (±SD) | 36.03 (0.89) | 35.84 (0.67) |
| V#2 Heart rate b/m, mean (±SD) | 78.37 (9.53) | 84.06 (12.60) |
| V#2 Oxygen saturation %, mean (±SD) | 95.00 (1.56) | 94.06 (3.17) |
| V#2 Axillary temperature °C, mean (±SD) | 35.82 (0.93) | 35.99 (0.95) |

**Table S2.** Baseline characteristics in patients of 65 years old or older.

SD: standard deviation; Kg: kilograms; μg/Kg/day: micrograms/kilogram/day; COPD: chronic obstructive pulmonary disease; COVID-19: coronavirus disease 19; IQR: interquartile range; ACEI: angiotensin converting enzyme inhibitors; ARB: angiotensin receptor blockers; g/dL: grams/deciliter; μL: microliter; mg/dL: milligrams/deciliter; g/L: grams/liter; U/L: units/liter; V#: visit number; b/m: beats/minute; °C: Celsius degrees

**Figure S11.** Hospitalization according to age

**Sub analysis by symptoms**

**Figure S12.** Distribution of patients according to symptoms

| Variables | Ivermectin (N=10) | Placebo (N=10) |
| --- | --- | --- |
| Demographic characteristics |  | |
| Age years, mean (±SD) | 56.04 (16.83) | 50.78 (16.98) |
| Weight Kg, mean (±SD) | 85.300 (14.182) | 83.000 (11.537) |
| Dose μg/Kg/day, mean (±SD) | 190.22 (19.72) | 187.50 (17.05) |
| Women % | 50.0 (5) | 60.0 (6) |
| Hypertension % | 60.0 (6) | 20.0 (2) |
| Diabetes mellitus % | 10.0 (1) | 20.0 (2) |
| Smoker % | 0.0 (0) | 10.0 (1) |
| Former smoker % | 50.0 (5) | 30.0 (3) |
| Asthma % | 10.0 (1) | 0.0 (0) |
| COPD % | 10.0 (1) | 0.0 (0) |
| Previous myocardial infarction % | 0.0 (0) | 10.0 (1) |
| Previous coronary angioplasty % | 0.0 (0) | 0.0 (0) |
| Previous stroke % | 0.0 (0) | 0.0 (0) |
| Heart failure % | 0.0 (0) | 0.0 (0) |
| Cancer % | 10.0 (1) | 0.0 (0) |
| Previous cancer % | 20.0 (2) | 0.0 (0) |
| Symptoms / Swabs |  | |
| Days from inclusion to swab 1, mean (±SD) | 2.89 (0.78) | 3.60 (0.70) |
| Days from inclusion to swab 2, mean (±SD) | 10.44 (1.67) | 11.20 (1.32) |
| Previous treatment |  | |
| Beta blockers % | 0.0 (0) | 10.0 (1) |
| ACEI % | 10.0 (1) | 10.0 (1) |
| ARB % | 30.0 (3) | 10.0 (1) |
| Aspirin % | 0.0 (0) | 10.0 (1) |
| Statins % | 0.0 (0) | 10.0 (1) |
| Puff inhalation % | 20.0 (2) | 0.0 (0) |
| Corticosteroids % | 20.0 (2) | 0.0 (0) |
| Laboratory values |  | |
| Hematocrit %, mean (±SD) | 43.70 (2.79) | 42.40 (5.15) |
| Hemoglobin g/dL, mean (±SD) | 14.40 (1.25) | 13.98 (1.82) |
| White blood count, mean (±SD) | 7270.00 (1724.79) | 5365.00 (2787.39) |
| Platelets /μL, mean (±SD) | 246500.00 (63444.20) | 250600.00 (103280.85) |
| Creatinine mg/dL, mean (±SD) | 0.89 (0.16) | 0.79 (0.31) |
| Urea g/L, median (IQR) | 0.31 (0.27-0.47) | 0.24 (0.23-0.28) |
| AST U/L, median (IQR) | 26 (20-32) | 38.5 (19-53) |
| ALT U/L, median (IQR) | 27 (13-40) | 37.5 (20-67) |
| Alkaline phosphatase U/L, median (IQR) | 210 (152-253) | 202 (170-262) |
| Total bilirrubin mg/dL, median (IQR) | 0.375 (0.30-0.40) | 0.225 (0.30-0.40) |
| Vital signs |  | |
| V#1 Heart rate b/m, mean (±SD) | 85.70 (16.41) | 83.20 (13.04) |
| V#1 Oxygen saturation %, mean (±SD) | 94.90 (7.11) | 95.50 (2.72) |
| V#1 Axillary temperature °C, mean (±SD) | 36.21 (0.56) | 35.92 (0.61) |
| V#2 Heart rate b/m, mean (±SD) | 78.78 (10.52) | 90.29 (14.35) |
| V#2 Oxygen saturation %, mean (±SD) | 96.44 (1.42) | 95.86 (2.54) |
| V#2 Axillary temperature °C, mean (±SD) | 35.64 (0.88) | 36.29 (0.50) |

**Table S3.** Baseline characteristics in asymptomatic patients.

SD: standard deviation; Kg: kilograms; μg/Kg/day: micrograms/kilogram/day; COPD: chronic obstructive pulmonary disease; COVID-19: coronavirus disease 19; IQR: interquartile range; ACEI: angiotensin converting enzyme inhibitors; ARB: angiotensin receptor blockers; g/dL: grams/deciliter; μL: microliter; mg/dL: milligrams/deciliter; g/L: grams/liter; U/L: units/liter; V#: visit number; b/m: beats/minute; °C: Celsius degrees

| Variables | Ivermectin (N=240) | Placebo (N=241) |
| --- | --- | --- |
| Demographic characteristics |  | |
| Age years, mean (±SD) | 42.14 (15.11) | 42.05 (15.64) |
| Weight Kg, mean (±SD) | 81.559 (18.674) | 81.244 (18.512) |
| Dose μg/Kg/day, mean (±SD) | 192.44 (24.77) | 191.74 (23.98) |
| Women % | 44.2 (106) | 49.8 (120) |
| Hypertension % | 19.7 (47) | 26.6 (64) |
| Diabetes mellitus % | 8.3 (20) | 10.5 (25) |
| Smoker % | 11.2 (27) | 10.0 (24) |
| Former smoker % | 28.0 (67) | 28.2 (68) |
| Asthma % | 6.3 (15) | 8.3 (20) |
| COPD % | 2.5 (6) | 2.9 (7) |
| Previous myocardial infarction % | 1.2 (3) | 2.1 (5) |
| Previous coronary angioplasty % | 1.2 (3) | 0.4 (1) |
| Previous stroke % | 0.4 (1) | 1.7 (4) |
| Heart failure % | 0.4 (1) | 1.2 (3) |
| Cancer % | 1.2 (3) | 0.8 (2) |
| Previous cancer % | 1.7 (4) | 1.7 (4) |
| Symptoms / Swabs |  | |
| Days from symptoms started to inclusion, median (IQR) | 4 (3-5) | 4 (3-6) |
| Days from inclusion to swab 1, mean (±SD) | 3.27 (0.65) | 3.28 (0.71) |
| Days from inclusion to swab 2, mean (±SD) | 9.97 (1.56) | 10.09 (1.70) |
| Previous treatment |  | |
| Beta blockers % | 7.1 (17) | 8.3 (20) |
| ACEI % | 5.0 (12) | 6.2 (15) |
| ARB % | 10.4 (25) | 14.5 (35) |
| Aspirin % | 5.8 (14) | 7.5 (18) |
| Statins % | 9.2 (22) | 6.2 (15) |
| Puff inhalation % | 3.3 (8) | 4.6 (11) |
| Corticosteroids % | 4.2 (10) | 4.6 (11) |
| Laboratory values |  | |
| Hematocrit %, mean (±SD) | 44.67 (5.19) | 43.33 (4.90) |
| Hemoglobin g/dL, mean (±SD) | 14.81 (1.81) | 14.33 (1.73) |
| White blood count, mean (±SD) | 5875.08 (1944.88) | 5558.71 (1731.97) |
| Platelets /μL, mean (±SD) | 232012.55 (66487.53) | 220841.67 (62400.78) |
| Creatinine mg/dL, mean (±SD) | 0.79 (0.25) | 0.81 (0.28) |
| Urea g/L, median (IQR) | 0.30 (0.24-0.37) | 0.30 (0.24-0.38) |
| AST U/L, median (IQR) | 27 (21-40) | 27 (20-41) |
| ALT U/L, median (IQR) | 31 (18-51) | 28 (18-51) |
| Alkaline phosphatase U/L, median (IQR) | 187 (150-234) | 186 (153-243) |
| Total bilirrubin mg/dL, median (IQR) | 0.30 (0.20-0.43) | 0.30 (0.20-0.42) |
| Vital signs |  | |
| V#1 Heart rate b/m, mean (±SD) | 83.07 (13.54) | 82.25 (13.46) |
| V#1 Oxygen saturation %, mean (±SD) | 96.14 (2.90) | 96.34 (1.97) |
| V#1 Axillary temperature °C, mean (±SD) | 36.15 (0.85) | 36.10 (0.81) |
| V#2 Heart rate b/m, mean (±SD) | 82.19 (12.73) | 83.07 (13.29) |
| V#2 Oxygen saturation %, mean (±SD) | 96.11 (2.31) | 96.04 (2.43) |
| V#2 Axillary temperature °C, mean (±SD) | 36.00 (0.74) | 36.03 (0.77) |

**Table S4.** Baseline characteristics in symptomatic patients.

SD: standard deviation; Kg: kilograms; μg/Kg/day: micrograms/kilogram/day; COPD: chronic obstructive pulmonary disease; COVID-19: coronavirus disease 19; IQR: interquartile range; ACEI: angiotensin converting enzyme inhibitors; ARB: angiotensin receptor blockers; g/dL: grams/deciliter; μL: microliter; mg/dL: milligrams/deciliter; g/L: grams/liter; U/L: units/liter; V#: visit number; b/m: beats/minute; °C: Celsius degrees

**Figure S13.** Hospitalization in patients symptomatic and asymptomatic

**Sub analysis by length of symptoms**

**Figure S14.** Distribution in patients symptomatic according to length of symptoms

| Variables | Ivermectin (N=209) | Placebo (N=198) |
| --- | --- | --- |
| Demographic characteristics |  | |
| Age years, mean (±SD) | 41.95 (14.84) | 42.34 (15.53) |
| Weight Kg, mean (±SD) | 82.015 (18.263) | 81.720 (18.407) |
| Dose μg/Kg/day, mean (±SD) | 191.67 (24.84) | 190.67 (24.43) |
| Women % | 43.1 (90) | 43.9 (87) |
| Hypertension % | 17.8 (37) | 26.3 (52) |
| Diabetes mellitus % | 8.6 (18) | 9.7 (19) |
| Smoker % | 11.5 (24) | 9.6 (19) |
| Former smoker % | 29.3 (61) | 30.3 (60) |
| Asthma % | 5.8 (12) | 7.1 (14) |
| COPD % | 2.4 (5) | 2.5 (5) |
| Previous myocardial infarction % | 1.4 (3) | 1.5 (3) |
| Previous coronary angioplasty % | 1.4 (3) | 0.0 (0) |
| Previous stroke % | 0.5 (1) | 1.5 (3) |
| Heart failure % | 0.5 (1) | 1.0 (2) |
| Cancer % | 1.4 (3) | 1.0 (2) |
| Previous cancer % | 1.9 (4) | 1.5 (3) |
| Symptoms / Swabs |  | |
| Days from symptoms started to inclusion, median (IQR) | 4 (2-5) | 3 (2-5) |
| Days from inclusion to swab 1, mean (±SD) | 3.26 (0.65) | 3.25 (0.66) |
| Days from inclusion to swab 2, mean (±SD) | 9.92 (1.54) | 10.16 (1.71) |
| Previous treatment |  | |
| Beta blockers % | 6.2 (13) | 8.6 (17) |
| ACEI % | 4.3 (9) | 5.1 (10) |
| ARB % | 9.6 (20) | 16.2 (32) |
| Aspirin % | 6.2 (13) | 7.6 (15) |
| Statins % | 9.1 (19) | 7.1 (14) |
| Puff inhalation % | 2.9 (6) | 3.5 (7) |
| Corticosteroids % | 4.8 (10) | 4.5 (9) |
| Laboratory values |  | |
| Hematocrit %, mean (±SD) | 44.82 (5.31) | 43.55 (5.13) |
| Hemoglobin g/dL, mean (±SD) | 14.84 (1.85) | 14.42 (1.82) |
| White blood count, mean (±SD) | 5894.88 (2024.22) | 5433.40 (1725.02) |
| Platelets /μL, mean (±SD) | 229043.27 (64853.63) | 216294.42 (55766.04) |
| Creatinine mg/dL, mean (±SD) | 0.79 (0.26) | 0.83 (0.29) |
| Urea g/L, median (IQR) | 0.30 (0.24-0.38) | 0.30 (0.24-0.39) |
| AST U/L, median (IQR) | 26 (21-38.5) | 27 (20-38.5) |
| ALT U/L, median (IQR) | 28.5 (18-51) | 27 (18-47) |
| Alkaline phosphatase U/L, median (IQR) | 187 (145.5-233.5) | 185 (154-239) |
| Total bilirrubin mg/dL, median (IQR) | 0.30 (0.20-0.405) | 0.30 (0.20-0.40) |
| Vital signs |  | |
| V#1 Heart rate b/m, mean (±SD) | 82.67 (13.66) | 81.16 (12.32) |
| V#1 Oxygen saturation %, mean (±SD) | 96.16 (3.05) | 96.28 (2.05) |
| V#1 Axillary temperature °C, mean (±SD) | 36.17 (0.87) | 36.13 (0.81) |
| V#2 Heart rate b/m, mean (±SD) | 82.32 (12.73) | 83.76 (13.65) |
| V#2 Oxygen saturation %, mean (±SD) | 96.10 (2.36) | 95.93 (2.53) |
| V#2 Axillary temperature °C, mean (±SD) | 36.02 (0.74) | 36.05 (0.79) |

**Table S5.** Baseline characteristics in patients with less than 7 days symptoms.

SD: standard deviation; Kg: kilograms; μg/Kg/day: micrograms/kilogram/day; COPD: chronic obstructive pulmonary disease; COVID-19: coronavirus disease 19; IQR: interquartile range; ACEI: angiotensin converting enzyme inhibitors; ARB: angiotensin receptor blockers; g/dL: grams/deciliter; μL: microliter; mg/dL: milligrams/deciliter; g/L: grams/liter; U/L: units/liter; V#: visit number; b/m: beats/minute; °C: Celsius degrees

| Variables | Ivermectin (N=31) | Placebo (N=43) |
| --- | --- | --- |
| Demographic characteristics |  | |
| Age years, mean (±SD) | 43.43 (16.98) | 40.69 (16.26) |
| Weight Kg, mean (±SD) | 78.484 (21.316) | 79.051 (19.055) |
| Dose μg/Kg/day, mean (±SD) | 197.80 (24.02) | 191.07 (22.09) |
| Women % | 52.0 (16) | 77.0 (33) |
| Hypertension % | 32.0 (10) | 28.0 (12) |
| Diabetes mellitus % | 6.0 (2) | 14.0 (6) |
| Smoker % | 10.0 (3) | 12.0 (5) |
| Former smoker % | 19.0 (6) | 19.0 (8) |
| Asthma % | 10.0 (3) | 14.0 (6) |
| COPD % | 3.0 (1) | 5.0 (2) |
| Previous myocardial infarction % | 0.0 (0) | 5.0 (2) |
| Previous coronary angioplasty % | 0.0 (0) | 2.0 (1) |
| Previous stroke % | 0.0 (0) | 2.0 (1) |
| Heart failure % | 0.0 (0) | 2.0 (1) |
| Cancer % | 0.0 (0) | 0.0 (0) |
| Previous cancer % | 0.0 (0) | 2.0 (1) |
| Symptoms / Swabs |  | |
| Days from symptoms started to inclusion, median (IQR) | 8 (7-10) | 8 (7-9) |
| Days from inclusion to swab 1, mean (±SD) | 3.35 (0.66) | 3.43 (0.89) |
| Days from inclusion to swab 2, mean (±SD) | 10.30 (1.70) | 9.74 (1.60) |
| Previous treatment |  | |
| Beta blockers % | 13.0 (4) | 7.0 (3) |
| ACEI % | 10.0 (3) | 12.0 (5) |
| ARB % | 16.0 (5) | 7.0 (3) |
| Aspirin % | 1.0 (3) | 7.0 (3) |
| Statins % | 10.0 (3) | 2.0 (1) |
| Puff inhalation % | 6.0 (2) | 10.0 (4) |
| Corticosteroids % | 0.0 (0) | 5.0 (2) |
| Laboratory values |  | |
| Hematocrit %, mean (±SD) | 43.64 (4.28) | 42.33 (3.58) |
| Hemoglobin g/dL, mean (±SD) | 14.56 (1.54) | 13.96 (1.19) |
| White blood count, mean (±SD) | 5742.90 (1313.66) | 6132.79 (1665.00) |
| Platelets /μL, mean (±SD) | 251935.48 (74681.96) | 241674.42 (84284.87) |
| Creatinine mg/dL, mean (±SD) | 0.74 (0.20) | 0.72 (0.23) |
| Urea g/L, median (IQR) | 0.28 (0.20-0.33) | 0.28 (0.22-0.35) |
| AST U/L, median (IQR) | 34 (24-47) | 26 (21-43) |
| ALT U/L, median (IQR) | 40 (29-51) | 40 (18-65) |
| Alkaline phosphatase U/L, median (IQR) | 191.5 (168.5-238.5) | 201 (152-252) |
| Total bilirrubin mg/dL, median (IQR) | 0.40 (0.20-0.60) | 0.30 (0.23-0.50) |
| Vital signs |  | |
| V#1 Heart rate b/m, mean (±SD) | 85.80 (12.60) | 87.25 (17.10) |
| V#1 Oxygen saturation %, mean (±SD) | 96.00 (1.62) | 96.63 (1.54) |
| V#1 Axillary temperature °C, mean (±SD) | 36.00 (0.76) | 35.96 (0.81) |
| V#2 Heart rate b/m, mean (±SD) | 81.21 (12.96) | 84.56 (11.39) |
| V#2 Oxygen saturation %, mean (±SD) | 96.21 (1.93) | 96.57 (1.78) |
| V#2 Axillary temperature °C, mean (±SD) | 35.93 (0.79) | 35.92 (0.69) |

**Table S6.** Baseline characteristics in patients with equal to or greater than 7 days symptoms.

SD: standard deviation; Kg: kilograms; μg/Kg/day: micrograms/kilogram/day; COPD: chronic obstructive pulmonary disease; COVID-19: coronavirus disease 19; IQR: interquartile range; ACEI: angiotensin converting enzyme inhibitors; ARB: angiotensin receptor blockers; g/dL: grams/deciliter; μL: microliter; mg/dL: milligrams/deciliter; g/L: grams/liter; U/L: units/liter; V#: visit number; b/m: beats/minute; °C: Celsius degrees

**Figure S15.** Hospitalization in patients symptomatic according to length of symptoms
